# Hepatoprotective and antioxidant activities of *Dicranopteris linearis* leaf extract against paracetamol-induced liver intoxication in rats

**DOI:** 10.1080/13880209.2020.1764058

**Published:** 2020-06-01

**Authors:** Zainul Amiruddin Zakaria, Farah Hidayah Kamisan, Teh Lay Kek, Mohd. Zaki Salleh

**Affiliations:** a Department of Biomedical Science, Faculty of Medicine and Health Sciences, Universiti Putra Malaysia, Malaysia; b Integrative Pharmacogenomics Institute (iPROMISE), Universiti Teknologi MARA, Selangor, Malaysia

**Keywords:** Paracetamol intoxication, polyphenolics, flavonoids, rutin, quercetin

## Abstract

**Context:**

*Dicranopteris linearis* L. (Gleicheniaceae) leaves have been reported to exert hepatoprotective activity.

**Objective:**

The hepatoprotective and antioxidant effects of ethyl acetate partition of *D. linearis* (EADL) are investigated.

**Materials and methods:**

EADL was subjected to antioxidant and anti-inflammatory studies, and phytochemical analyses. *In vivo* study involved six groups (*n* = 6) of overnight fasted Sprague Dawley rats. The test solutions [10% DMSO (normal), 10% DMSO (negative), 200 mg/kg silymarin (positive) or EADL (50, 250 or 500 mg/kg)] were administered orally once daily for 7 consecutive days followed by oral vehicle (only for normal) or hepatotoxic induction using 3 g/kg paracetamol (PCM).

**Results:**

EADL exerted ≈ 90% radical scavenging effects based on the DPPH and superoxide anion radical scavenging assays, high antioxidant capacity in the oxygen radical absorbance capacity assay (≈ 555,000 units), high total phenolic content (≈ 350 mg GAE/100 g extract) (*p* < 0.05), but low anti-inflammatory effect. EADL also attenuated PCM-induced liver intoxication as indicated by reduced level of serum liver enzymes; increased activity of endogenous enzymatic antioxidant (superoxide dismutase – 8.3 vs. 4.0 U/g tissue; catalase – 119 vs. 52 U/g tissue) and; reduced level of lipid peroxidation marker (2.7 vs. 5.0 µM). Preliminary screening of EADL revealed the presence of saponins, tannins and flavonoids with further HPLC analysis demonstrating the presence of rutin and quercetin.

**Discussion and conclusion:**

EADL exerted hepatoprotective and antioxidant activities; thus, these data support the potential use of *D. linearis* as a new source for future hepatoprotective drug development.

## Introduction

Liver disease accounts for approximately 2 million deaths per year worldwide (Asrani et al. 2019) and drugs are one potential cause of liver disease. Although drug-induced liver disease is rare, it still represents a serious clinical problem due to its unpredictable nature and potentially fatal course. Drug-induced liver disease accounts for as much as 20% of acute liver failure in paediatric populations and a similar percentage of adults with acute liver failure. Its exact incidence is difficult to document but about 40,000 to 45,000 people may experience a drug-induced liver injury each year (Kirchain and Allen 2014). Through its normally functioning enzymes and processes, the liver often causes a drug to become toxic via a process known as bioactivation.

One clinically important drug that has been associated with liver injury is paracetamol (PCM). Paracetamol (PCM), (Tylenol^®^ in the USA) is the most commonly used over-the-counter analgesic and antipyretic; it can be bought without prescription in most countries despite being the commonest cause of acute liver failure in Western countries (Du et al. 2016). PCM-induced hepatotoxicity remains the most common cause of acute liver failure in many countries. PCM overdose can cause severe liver injury, liver necrosis (Hinson et al. 2010), and kidney damage (Saleem and Iftikhar 2019) in human beings and animals. Given the public concern caused by PCM hepatotoxicity, great efforts have been made to understand the mechanisms of its toxic effects. Many studies indicate that oxidative stress is involved in various PCM associated toxicity including PCM-induced liver disease (Li et al. 2015).

It is important to briefly highlight that PCM, at therapeutic doses, is metabolised by cytochrome P450 enzymes to form a minor but extremely reactive metabolite known as *N*-acetyl-*p*-benzoquinoneimine (NAPQI), but this metabolite is metabolised and detoxified by glutathione (GSH). However, in PCM overdose, cytochrome P450-catalyzed metabolic activation of PCM generates excessive formation of the reactive NAPQI, which in turn depletes cellular GSH, resulting in a condition known as oxidative stress due to an imbalance between oxidants and antioxidants (Du et al. 2016). This results in the covalent binding of NAPQI to tissue macromolecules in the biomembrane such as DNA, proteins, carbohydrates and lipids that leads to the oxidation of the lipid components and finally liver injury. Damage to the biomembranes causes several liver enzymes such as ALT, AST, as well as ALP, and molecules such as bilirubin that are initially found in the cytoplasm of hepatocytes to leak into the blood stream. Moreover, since PCM is also metabolised by P450 enzymes in microsomes, it was assumed that P450-mediated metabolism of PCM also generated ROS that contribute to liver damage. The body exhibits protective measures against ROS via enzymes compounds [(e.g., superoxide dismutase (SOD), catalase (CAT), and GSH peroxidase (GSH-Px)], as well as nonenzymatic compounds (e.g., tocopherol/vitamin E, β-carotene, ascorbate, and GSH) (Jafari et al. 2014). Previous investigations found that the alteration of antioxidant enzyme activities in PCM overdose-induced oxidative stress were associated with a depletion of GSH and an increase of lipid peroxidation, all of which can lead to oxidative stress and finally cell death (Kisaoglu et al. 2014).

As such, the United States Food and Drug Administration (USFDA) recommends *N*-acetyl cysteine (NAC), a known antioxidant, as the only therapeutic option for PCM-overdosed patients; however, this medication has its limitations including adverse effects and a narrow therapeutic window (Du et al. 2016).

The 21st century has seen a paradigm shift towards therapeutic evaluation of herbal products in liver disease models by carefully synergizing the strengths of the traditional system of medicine with that of the modern concept of evidence-based therapeutic screening, authentication and randomised placebo-controlled clinical trials to support clinical efficacy. In spite of the tremendous advances made, no important and safe hepatoprotective drug is available. Therefore, due importance has been given globally to develop primarily plant-based hepatoprotective medications that are effective against a range of liver disorders (Ilyas et al. 2016).

One of the medicinal plants that have been reported to exert protective effects against PCM-induced liver disease is *Dicranopteris linearis* L. (Gleicheniaceae). Known as ‘*Pokok Resam*’ in Malay folklore medicine, the leaves of *D. linearis*, have been reported to exert hepatoprotective activity. Earlier studies have shown that the chloroform (Shamsahal Din et al. 2012), methanol (Mamat et al. 2013) and aqueous (Ismail et al. 2014) extracts of *D. linearis* leaves attenuate PCM- as well as carbon tetrachloride (CCl_4_)-induced liver intoxication. Further study using the methanol extract of *D. linearis* leaves (MEDL) also revealed the extract potential to attenuate PCM- and CCl_4_-induced liver intoxication by enhancing the endogenous antioxidant enzymes system (Kamisan et al. 2014; Zakaria et al. 2017).

Since methanol has the ability to extract polar, non-polar, and intermediate polar bioactive compounds, MEDL might contain a mixture of bioactive compounds that were different in their polarity. The present study was designed to determine the ability of intermediate polar bioactive compounds which exert hepatoprotective activity (McGaw et al. 2013). To achieve this, MEDL was sequentially partitioned to obtain the petroleum ether, ethyl acetate and aqueous partitions, which represent partition with polar, intermediate polar or non-polar bioactive compounds. The ethyl acetate partition of MEDL (EADL) was then subjected to PCM-induced hepatotoxicity study in rats.

## Materials and methods

### Sample preparation and preparation of MEDL

The leaves of *D. linearis* were collected between February and March, 2012 from their natural habitat around Serdang, Selangor, Malaysia. A voucher specimen (SK 1987/11) has been earlier issued by a certified botanist (Dr. Mohd. Shamsul Khamis) and was deposited at the Herbarium of the Institute of Bioscience, Universiti Putra Malaysia (UPM) (Kamisan et al. 2014). The leaves were dried for approximately two weeks in an oven at 40 °C. The dried leaves were ground to a coarse powder using a mill machine.

Approximately 160 g of powdered leaves were soaked in absolute methanol (ratio of 1:2; w/v) for 72 h at room temperature. The extraction process was repeated two more times. The supernatant collected from each extraction was filtered sequentially using a cloth filter, cotton wool, and Whatman No. 1 filter papers and then pooled together before being evaporated under reduced pressure (204 mbar) at 40 °C using a vacuum rotary evaporator (Buchi Rotavapor^®^ R210/215, Switzerland). The concentrated extract was left to stand in an oven at 30 °C to remove traces of methanol until a constant weight of extract was obtained.

### Preparation of EADL

Approximately 2 g of oven-dried MEDL was dissolved in methanol in a separating funnel followed by the addition of distilled water (ratio 1:2; v/v). The mixture was shaken vigorously to enable complete mixture. The mixture was then mixed with 700 mL of petroleum ether, shaken vigorously and then left for 2 h to settle into two-phase immiscible liquid solutions before the lower phase was collected. These procedures were carried out three times. The collected petroleum ether supernatant was pooled together and then subjected to the evaporation process. The upper layer was mixed with 700 mL of ethyl acetate, shaken vigorously and then left for 2 h to settle into two-phase immiscible liquid solutions before the lower phase was collected. These procedures were repeated three times. The collected ethyl acetate supernatant was also pooled together and then subjected to the evaporation process. Finally, following the partitioning with ethyl acetate, the remaining residue left was collected as the aqueous partition of MEDL, which was evaporated to remove methanol residue and then kept at −80 °C for at least 48 h before subjected to the freeze-drying process. These partitioning methods were adopted from Ismail Suhaimy et al. (2017) with modifications.

### Evaluation of antioxidant activity of EADL

#### Total phenolic content (TPC) of EADL

Total phenolic content (TPC) of EADL was determined using the Folin-Ciocalteu procedure with minor alterations as described by Yahya et al. (2013). Briefly, 1 mg/mL of methanolic solution of EADL was extracted at room temperature with 1 mL of 80% methanol mixed with 1% hydrochloric acid and 1% distilled water on the shaker set at 200 rpm for 2 h. After that, the mixture was centrifuged at 2817 *g* for 15 min and the supernatant obtained was decanted into vials. The reaction mixture was later prepared by mixing 200 μL of EADL supernatant with 400 μL (0.1 mL/0.9 mL) of Folin-Ciocalteu reagent and allowed the mixture to stand at room temperature for 5 min. Then, 400 μL sodium bicarbonate (60 mg/mL) solution was added and the mixture was again allowed to stand at room temperature for 90 min. The absorbance was determined using a spectrophotometer at 725 nm. A calibration curve was generated by using the gallic acid (GA) standard optical density, and the levels in the samples were expressed in terms of mg GA equivalent (GAE)/100 g extract. Based on the measured absorbance, the concentration of phenolics was read (mg/mL) from the calibration line.

#### 2,2-Diphenyl-1-picrylhydrazyl (DPPH) radical scavenging assay

The ability of EADL to induce free radicals scavenging action was determined using the 2,2-diphenyl-1-picrylhydrazyl (DPPH) assay with slight modifications as described by Yahya et al. (2013). Approximately 50 μL of EADL (1 mg/mL) was mixed with 50 μL DPPH (FG: 384.32) (1 mM in ethanolic solution) and 150 μL absolute ethanol (AR grade) in a 96-well microtiter plate in triplicate. The reaction mixture contained test sample ranging in the concentration of 3.13 to 200 µg/mL. The 96-well microtiter plate was shaken for 15 sec at 500 rpm and then left to stand at room temperature for 30 min before the absorbance was recorded at 520 nm using the Schimadzu UV-Vis 1700 spectrophotometer. The DPPH radical concentration was calculated using the following equation:

$$\text{DPPH}\;\text{scavenging}\;\text{effect}\;\left(\%\right)=\left[\left(A_{0}–A_{x}\right)/\left(A_{0}–A_{1}\right)\right]\text{ × }100$$

where A_0_ is absorbance of negative control, A_1_ is the absorbance of positive control (*L*-ascorbic acid) and Axe is the absorbance of sample. The IC_50_ was described as the amount of the sample that is sufficient to elicit 50% reduction of the initial DPPH radical concentration. This was calculated from the linear part of the inhibition of DPPH radical (Nithianantham et al. 2011). Data analysis was done by using Graph pad PRISM V5.01.

#### Superoxide anion radical scavenging assay

The ability of EADL to induce the superoxide anion radicals scavenging action was measured according to the slightly modified method described by Liu et al. (1997). Briefly, the superoxide radicals were produced in phenazine methosulphate-nicotinamide adenine dinucleotide (PMS-NADH) systems via oxidation of NADH and evaluated by the reduction of nitroblue tetrazolium (NBT). In these experiments, the superoxide radicals were produced in 3 mL of Tris-HCl buffer (16 mM, pH 8) containing 1 mL of NBT (50 μM), 1 mL NADH (78 μM) and EADL (25–50 μg). The reaction was started by adding 1 mL of PMS solution (10 μM) to the mixture. The reaction mixture was incubated at 25 °C for 5 min and then the absorbance was read at 560 nm using a spectrophotometer (Schimadzu UV-Vis 1700) against blank samples using *L*-ascorbic acid as a positive control. Decreased absorbance of the reaction mixture indicated increasing superoxide anion scavenging activity. The percentage inhibition of superoxide anion production was evaluated using the following formula:

$$\text{Inhibition}\;\text{of}\;\text{superoxide}\;\text{anion}\;\text{generation}\;\left(\%\right)=\left[1-\left(A_{T}/A_{C}\right)\right]\times 100$$

where A_C_ was the absorbance of the control (SOD), and A_T_ was the absorbance in the presence of EADL or standard.

#### Oxygen radical absorbance capacity (ORAC) test

The antioxidant capacity of EADL was also determined using the slightly modified oxygen-radical absorbance-capacity (ORAC) assay as described by Huang et al. (2002). Briefly, 2,2-azobis (2-amidinopropane) dihydrochloride (AAPH) was dissolved in 10 mL of 75 mM phosphate buffer (pH 7.4), which was prepared daily as peroxyl-radical generator. A fluorescein stock solution (1 mM) was prepared in 75 mM phosphate buffer (pH 7.4) and stored in wrapping foil at 5 °C. During the analysis, the sodium fluorescein stock solution was diluted 1:100 000 with 75 mM phosphate buffer (pH 7.4). The microplate wells were filled with 150 μL of working solution of sodium fluorescein and 25 μL of Trolox (positive control) dilution in blank wells or with 25 μL of EADL (200 μg/mL) in sample wells and then was equilibrated by incubating for 10 min at 37 °C. After the solutions equilibrated, 25 μL of 240 mM AAPH solution was added to the wells to initiate the reactions. BMG Omega Fluostar Fluorescent Spectrophotometer (BMG LABTECH, Ortenberg, Germany) with injector was used with an excitation filter of 485 nm and an emission filter of 520 nm. The fluorescence intensity of each well was then measured kinetically with data taken every 1 min for 3 h. ORAC values were calculated using MARS Data Analysis Reduction Software.

### Evaluation of anti-inflammatory activity of EADL

#### Lipoxygenase (LOX) assay

The lipoxygenase (LOX) assay was carried out using the slightly modified spectrophotometric method as described by Azhar-Ul-Haq et al. (2004). Approximately 160 μL of sodium phosphate buffer (0.1 M, pH 8.0), 10 μL of EADL and 20 μL of soybean LOX solution were mixed and then incubated for 10 min at 25 °C. About 10 μL of the substrate in the form of sodium linoleic acid solution was added to initiate the reaction. The enzymatic conversion of linoleic acid to form (9Z,11E)-(13S)-13-hydroperoxyoctadeca-9,11-dienoate was followed by the measurement of absorbance change at 234 nm over the period of 6 min. Reference standards and EADL were dissolved in methanol. All reactions were completed in triplicates in a 96-well microplate.

#### Xanthine oxidase (XO) assay

The spectrophotometric method described by Noro et al. (1983) was adopted to measure the xanthine oxidase (XO) inhibiting activity of EADL with slight modifications. Approximately 10 μL of the test solution and 10 μL of XO solution were mixed with 130 μL of potassium phosphate buffer (0.05 M, pH 7.5) and incubated for 10 min at 25 °C. Then 100 μL of the substrate in the form of xanthine solution was added to initiate the reaction. The enzymatic conversion of xanthine to form uric acid and hydrogen peroxides was followed by the measurement of absorbance change at 295 nm. Reference standard and EADL were dissolved in DMSO. All reactions were completed in triplicates in a 96-well microplate.

#### Experimental animals

Male *Sprague Dawley* rats obtained from the Animal Source Unit, Faculty of Veterinary Medicine (FVM), Universiti Putra Malaysia (UPM), Selangor, Malaysia were used in the hepatoprotective study. The rats (180 to 200 g; 8 to 10 weeks old) were housed in spacious, hygienic polyethylene cages with wood shaving bedding that were maintained at a constant 27 ± 2 °C temperature with 70–80% humidity on a standard 12 h light/dark cycle in the Animal House Unit, Faculty of Medicine and Health Sciences, UPM, for at least 48 h before use. Food and water were available *ad libitum* up to the beginning of the experiments. The animals were handled in compliance with current UPM guidelines for the care of laboratory animals and the ethical guidelines for investigations of experimental pain in conscious animals. The study protocol of the present study was approved by the Animal House and Use Committee, Faculty of Medicine and Health Sciences, UPM (Ethical approval no.: UPM/FPSK/PADS/BR-UUH/00449).

#### Hepatoprotective assay

Rats were divided into six groups with equal number of rats (*n* = 6) through random selection and were fasted overnight. Group 1, served as the normal control, was pre-treated orally with vehicle 1 (10% DMSO) for 7 days before being treated orally with vehicle 2 (10% DMSO); Group 2, served as the hepatotoxic group, was pre-treated orally with vehicle for 7 days followed by the oral administration of 3 mg/kg PCM, which was suspended in 10% DMSO; Groups 3, served as the standard drug group, received (oral) 200 mg/kg silymarin, which was suspended in 1% carboxymethyl cellulose (CMC). Group 4, 5 and 6 received EADL, in the respective concentration of 50, 250 or 500 mg/kg, via oral administration for 7 consecutive days before subjected to the PCM-induced hepatotoxic study. Prior to the administration of test solutions, each group of rats were weighed daily within the period of hepatoprotective study. Each group of rats received the respective dose of test solutions orally once daily for 7 consecutive days. Three hours after the administration of pre-treatment drugs on day 7^th^, Groups 3, 4, 5, 6, 7, 8 and 9 were given PCM. The experimental groups are summarised as in Table 1.

**Table 1.** Experimental design on hepatoprotective effect of EADL against PCM-induced rats.

Group	Type of treatment	Pre-treatment^a^	Dose (mg/kg)	Hepatotoxic inducer^b^
1	Normal control	10% DMSO	–	10% DMSO
2	Hepatotoxic control	10% DMSO	–	3 mg/kg PCM
3	Positive control	Silymarin	200 mg/kg	3 mg/kg PCM
4	Treatment	EADL	50 mg/kg	3 mg/kg PCM
5	Treatment		250 mg/kg	3 mg/kg PCM
6	Treatment		500 mg/kg	3 mg/kg PCM

^a^Administered orally and daily for 7 consecutive days.

^b^Administered orally 3 h after the last pre-treatment on 7^th^ day.

All test solutions were administered in the volume of 10 ml/kg.

Forty-eight hours after the administration of PCM, rats from each of the group were anaesthetised using intramuscularly-administered 100 mg/kg ketamine and 16 mg/kg xylazine. Blood collection was then performed on each rat via cardiac puncture into heparinised bottles and, then, the rats were euthanized by cervical dislocation. Upon euthanization, the liver was instantaneously removed, washed in ice-cold saline to remove blood and duly weighed. A section from the median lobe of the liver was preserved in 10% formalin solution for histological analysis. The remaining liver was quickly frozen in dry ice and stored at −80 °C for further endogenous antioxidant enzymes analysis.

#### Biochemical analysis

The blood was centrifuged at 3000 rpm for 15 min to obtain the plasma, which was then aspirated off and frozen at −80 °C. The plasma samples were later analysed to determine the level of alanine aminotransferase (ALT), alkaline phosphatase (ALP), aspartate aminotransferase (AST), and total bilirubin according to standard methods and measured using a Hitachi 902 Automatic Chemical Analyser.

#### Determination of antioxidant enzymes level and MDA level in liver homogenates

A 10% (w/v) homogenate, obtained from homogenisation of liver tissue in cold phosphate buffer (5 mM, pH 7.4), was centrifuged at 4000 rpm for 15 min at 4 °C. Protein concentration was determined using the Bradford method as described by Lowry et al. (1951) with bovine serum albumin (BSA) used as a standard. Then, the supernatant of liver homogenate was subjected to the estimation of SOD and CAT activity and MDA level using the commercial kits according to the respective manufacturer’s instructions (Superoxide Dismutase Assay Kit, Catalase Assay Kit and TBARS Assay Kit, Cayman Chemical Company, Ann Arbor, MI, USA).

#### Histopathological analysis of PCM-intoxicated liver tissue

The livers were regularly processed using the Automatic Tissue Processor (Leica TP1020, Germany), and then embedded in paraffin wax with Leica EG 1160 (Leica Microsystems, Germany). Each tissue was sectioned to a thickness between 5 to 6 µm using a microtome (Leica RM2125 RTS, Singapore) and then stained with haematoxylin and eosin dye using the Tissue-Tek Prisma-Ezs Autostainer (Sakura, Torrance, CA, USA) for microscopic observation of histopathological changes in the livers. The microscopic observation was done by using light microscope Olymphus-CX31 (Olympus, Japan). The liver sections were then scored and evaluated by pathologist according to the severity of the hepatic injury as described by El-Beshbishy et al. (2010) with modifications.

#### Phytochemical screening of EADL

EADL was investigated by qualitative tests for saponins, steroids, flavonoids, triterpenes, tannins and alkaloids according to the detailed procedures described by Ikhiri et al. (1992).

#### High performance liquid chromatography (HPLC) analysis of EADL

EADL was subjected to HPLC analysis using the following HPLC system; Waters Delta 600 with 600 Controller equipped with Waters 996 photodiode array detector and a Phenomenex Luna column (5 µm; 4.6 mm i.d. × 250 mm) (Torrance, CA, USA). The procedures used were as reported by Kamisan et al. (2014). In brief, two types of eluants, namely, 0.1% aqueous formic acid (eluant A) and acetonitrile (eluant B) were used. The gradient elution was programmed with the initial condition of 95% A and 5% B (acetonitrile) with a linear gradient reaching 25% B at *t* = 12 min. After 10 min (*t* = 22 min), gradient of B was decreased to 15% and sustained for 12 min (*t* = 30 min). Starting from *t* = 30 min, the programme was returned to the initial solvent composition at *t* = 35 min. Millennium 32 Chromatography Software (Waters Co., Milford, MA, USA) was used to record and integrate the chromatograms at peak areas with the peak elution monitored at various wavelengths, namely 210, 254, 280, 300, 330 and 366 nm. The verified chromatograms were analysed and the recorded retention times, peak areas and UV spectra of main peaks were studied. The HPLC profile of EADL was also analysed by comparing the chromatogram of EADL against the respective chromatograms of several pure bioactive compounds available in the laboratory, namely fisetin, quercetin rutin, quercitrin, naringenin, genistein, pinostrobin, hesperetin, flavanone, 4′,5,7-trihydroxyflavanone, 2,4,4′-trihydroxychalcone, dihydroquercetin, or hesperetin at 254 nm. These bioactive compounds were earlier purchased from the Sigma Aldrich Co. (St. Louis, MO, USA). Retention times and the UV spectrums for each of the peak of standard pure compounds were compared against the peaks of EADL and, the similarity indicate the presence of the specific bioactive compounds.

### Statistical analysis

Data were expressed as mean values ± SD of six rats in each group. Statistical analysis was performed using one-way analysis of variance (ANOVA) followed by Dunnet’s Multiple Comparison test with *p* < 0.05 being considered statistically significant. Statistical analysis was conducted with Graph Pad Prism software version 5.

## Results

### Extraction yield of EADL

Approximately 74.00 g of MEDL were partitioned to yield 3.38 g of PEDL, 8.59 g of EADL and 11.27 g of AQDL. EADL was further tested for antioxidant, anti-inflammatory, and hepatoprotective activities.

### Antioxidant activity of EADL

#### TPC value of EADL

The TPC value of EADL recorded after colorimetric analysis was approximately 352.18 ± 48.40 mg GAE/100 g extract (Table 2). This finding indicates that EADL contained a high TPC value and corresponds with the standard requirement that any compounds with TPC value greater than 100 mg GAE/100 mg extract is considered to have a high TPC value.

**Table 2.** TPC value, free radical scavenging activity and antioxidant capacity of EADL.

Sample	Concentration (µg/ml)	Standard drug	TPC value (mg GAE/1 g dry weight of extract)^a^	Free radical scavenging activity		Antioxidant capacity
				DPPH (% AA)	SOA (% AA)	ORAC^b^ (mmol TE/1 g dry weight of extract)
EADL	200	Gallic acid	352.2 ± 48.4	–	–	–
		Ascorbic acid	–	93.7 ± 3.0	–	–
			–	–	92.6 ± 2.2	–
		Trolox	–	–	–	555 ± 12.7

All values are expressed as mean ± SEM.

% AA refers to percentage of antioxidant potential.

^a^Data, expressed as TPC mg GAE/1 g dry weight of extract, were mean values of triplicate wells in duplicate experiments., Standard error of mean (SEM) < 5%.

^b^Data, expressed as ORAC value mmol Trolox Equivalent (TE)/1 g dry weight of extract, were mean values of triplicate wells in duplicate experiments, standard error mean (SEM) < 30%.

#### Effect of EADL against the DPPH- and SOA-radical scavenging activities, and ORAC value

Effect of EADL against the DPPH radical scavenging assay is depicted in Table 2. At the concentration of 200 µg/mL, EADL caused a 94% radical scavenging activity when compared to the standard drug, 200 µg/mL ascorbic acid. The IC_50_ value recorded for EADL was 76 µg/mL.

Effect of EADL against the SOA radical scavenging assay is also shown in Table 2. At the concentration of 200 µg/mL, EADL caused 93% radical scavenging activity when compared to the standard drug, 200 µg/mL ascorbic acid.

In addition, the antioxidant capacity of EADL measured using the ORAC assay is also demonstrated in Table 2. At the concentration of 200 µg/mL, EADL was found to produce approximately 555 mmol Trolox Equivalent (TE)/1 g dry weight of extract.

### *In vitro* anti- inflammatory activity of EADL

#### Effect of EADL against the LOX- and XO-mediated inflammatory assays

Effect of EADL on *in vitro* LOX- and XO-mediated inflammatory assay is shown in Table 3. At the concentration of 100 µg/mL, EADL induced a low inhibitory effect against both inflammatory enzymes activity with the recorded value of approximately 26% and 1.5%, respectively.

**Table 3.** Effect of EADL on *in vitro* inflammatory activity mediated by LOX and XO.

Sample	Concentration (µg/ml)	Inhibition of Inflammation (%)	
		LOX activity	XO activity
EADL	100	25.9 ± 4.4	1.5 ± 0.2

All values are expressed as mean ± SEM.

Note: H, high (71–100%); M, moderate (41–70%); L, low (0–40%); NA, not active (Ismail Suhaimy et al. 2017).

### *In vivo* hepatoprotective activity of EADL against PCM-induced intoxication

#### Effect of EADL pre-treatment on the PCM-intoxicated rat body weight (BW), liver weight (LW) and their ratio (LW/BW)

Table 4 shows the effect of EADL pre-treatment on body weight (BW), liver weight (LW) and their ratio (LW/BW) in PCM-intoxicated rats. Rats from PCM-induced hepatotoxic control group (Group 2) showed a significant (*p* < 0.05) increase in the LW when compared with the normal control group (Group 1). Pre-treatment with EADL, at all concentrations, significantly (*p* < 0.05) decreased the LW when compared to the PCM-intoxicated group. Interestingly, pre-treatment with EADL as well as 200 mg/kg silymarin also caused significant (*p* < 0.05) reduction in the LW/BW ratio, thus, indicating their ability to attenuate PCM-induced liver damage.

**Table 4.** Effect of EADL pre-treatment on the body weight (BW), liver weight (LW) and their ratio (LW/BW) in PCM intoxicated rats.

Group	Treatment	Dose (mg/kg)	Body weight (BW) (g)	Liver weight (LW) (g)	LW/BW (%)
Normal Control	Control	–	208.7 ± 5.6	5.9 ± 0.3	2.8 ± 0.1
Negative Control	DMSO + PCM*	–	219.5 ± 4.7	9.7 ± 0.9^a^	4.4 ± 0.4^a^
Positive Control	Silymarin + PCM*	200	209.4 ± 4.7	6.9 ± 0.2^b^	3.3 ± 0.2^b^
Treatment	EADL + PCM*	50	215.7 ± 6.6	7.1 ± 0.5^b^	3.3 ± 0.3^b^
		250	219.8 ± 7.1	7.8 ± 0.2^b^	3.6 ± 0.4^b^
		500	217.5 ± 4.7	6.2 ± 0.4^b^	2.9 ± 0.3^b^

Values are expressed as means ± S.E.M. of six replicates.

*PCM was administered in the dose of 3 mg/kg.

^a^Significant different as compared to normal control, *p* < 0.05.

^b^Significant different as compared to negative control, *p* < 0.05.

#### Effect of EADL pre-treatment on the serum biochemical parameters (ALT, AST, ALP and TB) in PCM-intoxicated rats

Table 5 depicts the effect of EADL pre-treatment on serum liver biochemical parameters, namely ALT, AST, ALP and TB, in PCM-intoxicated rats. Liver damage induced by PCM (Group 2) caused significant (*p* < 0.05) increased in serum liver marker enzymes (i.e., ALT, AST and ALP), and TB levels when compared to the normal control group (Group 1), which suggested that there was leakages of the said enzymes and TB into the blood circulation resulting from the liver damaged. Pre-treatment with EADL, at all concentrations, caused significant (*p* < 0.05) reduction in PCM-induced increase levels of ALT, AST, ALP and TB. This reversal against PCM-induced effects was also seen with silymarin (Group 3).

**Table 5.** Effect of EADL pre-treatment on the levels of ALT, AST, ALP, and TB in PCM intoxicated rats.

Group	Treatment	Dose (mg/kg)	ALT (U/L)	AST (U/L)	ALP (U/L)	TB (umol/L)
Normal control	Control	–	15.8 ± 2.9	95.1 ± 5.9	115.7 ± 7.0	0.5 ± 0.2
Negative control	DMSO + PCM*	–	1714.3 ± 142.2^a^	2266.2 ± 340.4^a^	330.0 ± 42.4^a^	4.1 ± 0.8^a^
Positive control	Silymarin + PCM*	200	474.5 ± 82.2^b^	690.9 ± 146.6^b^	195.5 ± 11.1^b^	2.3 ± 0.3^b^
Treatment	EADL + PCM*	50	1024.4 ± 230.1^b^	971.7 ± 226.1^b^	377.8 ± 53.7	1.1 ± 0.2^b^
		250	608.9 ± 340.0^b^	800.8 ± 291.0^b^	225.0 ± 10.0^b^	1.3 ± 0.4^b^
		500	108.7 ± 29.0^b^	313.0 ± 66.0^b^	198.5 ± 19.8^b^	1.5 ± 0.3^b^

Values are expressed as means ± S.E.M. of six replicates.

*PCM was administered in the dose of 3 mg/kg.

^a^Significant different as compared to normal control, *p* < 0.05.

^b^Significant different as compared to negative control, *p* < 0.05.

#### Effects of EADL pre-treatment on the activities of endogenous antioxidant enzymes defence (i.e., CAT and SOD) and the level of end product of lipid peroxidation (i.e., MDA)

Effect of EADL on the activities of several endogenous antioxidant enzymes defence namely CAT and SOD, is shown in Table 6. In the PCM-induced hepatotoxic group (Group 2), a significant (*p* < 0.05) decreased in the liver CAT and SOD activities was observed in comparison to the normal control group. However, pre-treatment with silymarin or EADL, at the doses of 250 and 500 mg/kg, was found to reverse the toxic effect of PCM by causing a significant (*p* < 0.05) increase in the activities of liver’s CAT and SOD when compared to the PCM-intoxicated group (Group 2) indicating the ability of EADL to trigger hepatoprotective activity partly via the activation of endogenous enzymatic antioxidant system.

**Table 6.** Effect of EADL pre-treatment on the levels of SOD, CAT and MDA in liver of PCM intoxicated rats.

Group	Treatment	Dose (mg/kg)	CAT (U/g tissue)	SOD (U/g tissue)	MDA (µM)
Normal control	DMSO + DMSO	–	114.8 ± 1.6	9.7 ± 0.4	2.6 ± 0.6
Negative control	DMSO + PCM*	–	92.9 ± 1.9^a^	4.0 ± 0.1^a^	5.0 ± 0.6^a^
Positive control	Silymarin + PCM*	200	109.5 ± 4.6^b^	11.8 ± 1.5	2.6 ± 0.3^b^
Treatment	EADL + PCM*	50	96.9 ± 2.7	5.3 ± 0.9	2.6 ± 0.7^b^
		250	112.7 ± 5.7^b^	7.9 ± 0.2^b^	3.0 ± 0.3^b^
		500	115.2 ± 3.4^b^	8.3 ± 0.5^b^	2.7 ± 0.7^b^

Values are expressed as means ± S.E.M. of six replicates.

*PCM was administered in the dose of 3 mg/kg.

^a^Significant different as compared to normal control, *p* < 0.05.

^b^Significant different as compared to negative control (DMSO + PCM), *p* < 0.05.

Table 6 also shows the effect of EADL pre-treatment on the level of end product of lipid peroxidation, namely MDA. Level of MDA in the liver of PCM intoxicated rats (Group 2) was significantly (*p* < 0.05) elevated in comparison to the normal control group (Group 1). Silymarin and all doses of EADL significantly (*p* < 0.05) reduced the toxic effect of PCM by reversing and restoring the level of MDA towards its normal value when compared to the PCM-intoxicated group (Group 2) indicating that EADL induced hepatoprotective activity partly via the attenuation of lipid peroxidation.

#### Histopathological findings on the PCM-induced hepatotoxic liver with or without EADL pre-treatment

**Figure 1.**
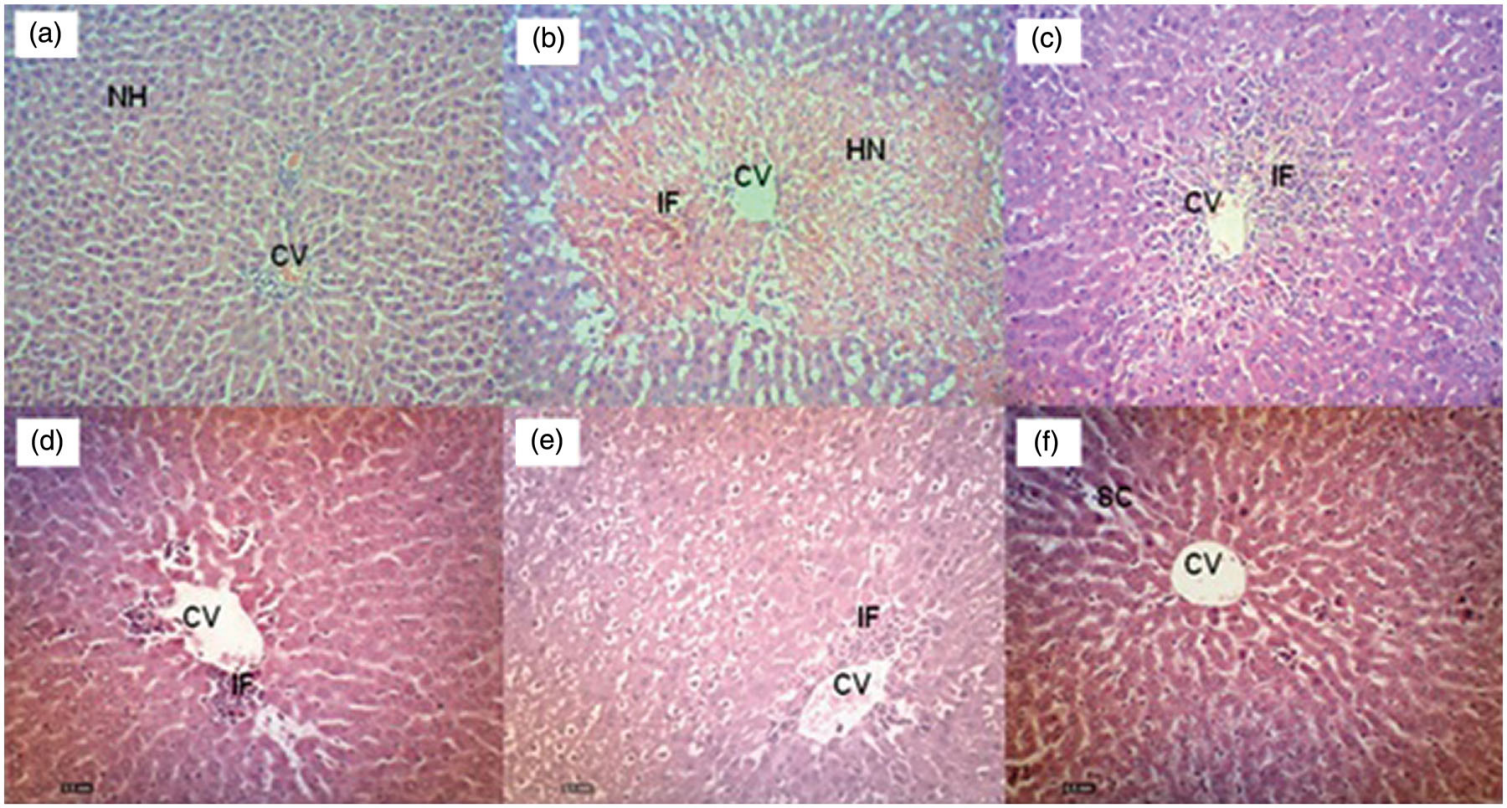
(a) Normal liver tissue, b) PCM intoxicated liver tissue (negative control) showed large area of haemorrhagic necrosis around centrilobular region, and inflammatory cell infiltration at the centre of the necrotic foci, c) Effect of 200 mg/kg silymarin pre-treatment on PCM intoxicated liver tissue showing preservation of normal hepatocytes, d) Effect of 50 mg/kg EADL pre-treatment on PCM intoxicated liver tissue showing mild sinusoidal congestion and cellular swelling, e) Effect of 250 mg/kg EADL pre-treatment on PCM intoxicated liver tissue showing moderate haemorrhagic necrosis in centrilobular region and presence of inflammatory infiltrate, f) Effect of 500 mg/kg EADL pre-treatment on PCM intoxicated liver tissue showing mild inflammatory infiltrate and mild cellular swelling. (H&E staining, 100x magnification). CV) Central vein. IF) Inflammatory infiltrate. HN) Haemorrhagic necrosis. SC) Sinusoidal congestion.

Effect of EADL pre-treatment on the PCM-intoxicated liver histopathology is presented in Figure 1. Histopathological observations of liver sections from the normal control group (Group 1) showed normal cellular architecture with distinct hepatic cells, sinusoidal spaces and a central vein (Figure 1(a)). In contrast, the PCM- intoxicated group (Group 2) exhibited the most severe damage of cellular architecture with massive necrosis, ballooning degeneration, broad infiltration of lymphocytes, and the loss of cellular boundaries observed (Figure 1(b)). Pre-treatment with 200 mg/kg silymarin (Group 3) reversed the effect of PCM intoxication on the liver indicated by changes that almost improved the cellular architecture such as the presence of only patches of necrotic hepatocytes (Figure 1(c)). On the other hand, pre-treatment with EADL, at the doses of 50, 250, and 500 mg/kg, caused a relatively normal lobular pattern with a mild degree of necrosis and lymphocyte infiltration when compared to the PCM-intoxicated group (Figure 1(d–f)). The histological scoring of those changes is provided in Table 7.

**Table 7.** Histopathological scoring of the liver section of PCM intoxicated rats with or without EADL pre-treatment.

Treatment	Dose (mg/kg)	Steatosis	Necrosis	Inflammation	Hemorrhage
Control	−	−	−	−	−
DMSO + PCM		+	+++	++	+
Silymarin + PCM	200	−	+	−	−
EADL + PCM	50	−	+	−	−
	250	+	++	+	−
	500	+	−	−	−

The severity of various features of hepatic injury was evaluated based on those following scoring scheme:

− normal; + mild effect; ++ moderate effect; +++ severe effect.

### Phytochemicals content of EADL

Following the qualitative phytochemical screening of EADL, several classes of bioactive compounds, namely saponins, flavonoids, tannins and polyphenolic compounds and steroids were detected in EADL (Table 8).

**Table 8.** Comparison on the presence of phytochemical constituents in different partitions of MEDL.

Sample	Phytochemical constituents						Conclusion
	Alkaloids	Saponins	Flavonoids	Tannins and Polyphenolic compound	Triterpenes	Steroids	
PEDL	−	1+	−	−	−	1+	Saponins and steroids only presence.
EADL	−	3+	1+	2+	−	2+	Saponins, flavonoids, tannins and polyphenolic compound and steroids presence.
AQDL	−	1+	−	−	1+	−	Saponins and triterpenes only presence.

### HPLC profile of AQDL

Figure 2(a) shows the HPLC profiles of EADL determined at 210, 254, 280, 300, 330, and 366 nm. From the chromatograms obtained, various peaks were detected at different wavelengths. Of these, only six major peaks were constantly detected at all wavelengths with the recorded retention time (R_T_) of 19.3 (Peak 1), 19.9 (Peak 2), 20.8 (Peak 3), 22.7 (Peak 4), 23.1 (Peak 5) and 26.8 (Peak 6) min (Figure 2(b)).

Figure 2.(a) HPLC profile of EADL determined as various wavelength (210–366 nm). (b) HPLC chromatogram of EADL. Various peaks were detected at different wavelengths with some of them found to possess UV-vis spectral that is a characteristic of flavonoid-based bioactive compound, namely peaks with a retention time (R_T_) of 19.3, 19.9, 20.8, 22.7, 23.1 and 26.8 min. (c) Comparison between the chromatogram of EADL against the chromatograms of eleven pure flavonoids revealed the presence of only rutin (*R*_T_ = 20.1 min) and quercetin (*R*_T_ = 26.8 min) in EADL. Only four pure flavonoid chromatograms were included for comparison with EADL.

**Figure:**
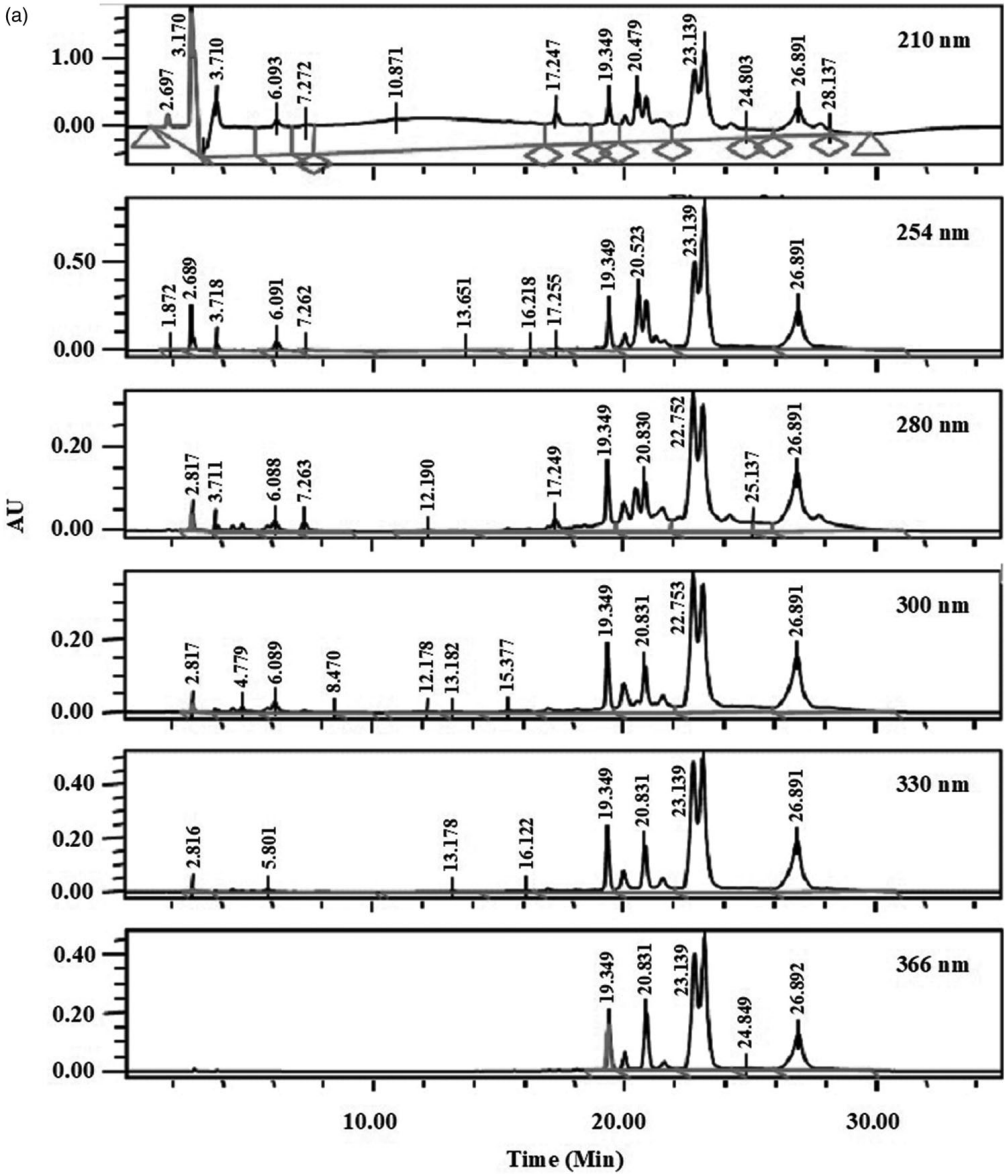

**Figure:**
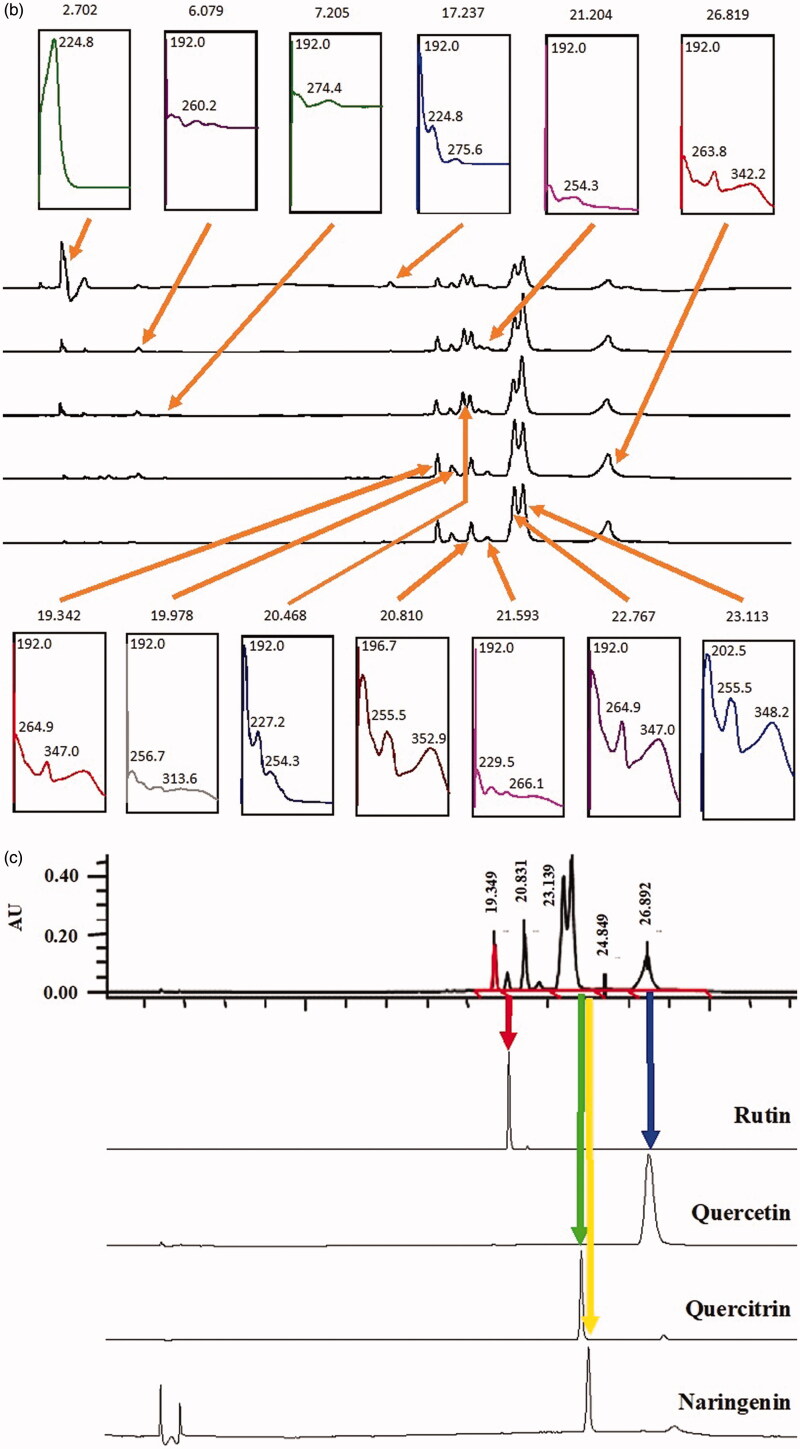

Further analysis of these six peaks based on their UV-vis spectra revealed that they represent bioactive compounds of flavonoids group (Table 9). Peaks 1, 2, 4, 5 and 6, which exerted the UV-vis spectra with two absorbance bands, namely Band A that lies in the 310–350 nm range and Band B that falls in the 250–290 nm range, represent bioactive compounds of the flavones type. On the other hand, Peak 3 has the UV-vis spectra with Band A that fall in the range of 350 and 385 nm and Band B that lies in the range of 250–290 nm, represents bioactive compound of the flavonol type.

**Table 9.** Peak with UV-vis spectra that was a characteristic of a flavonoid-based bioactive compounds.

Peak	R_T_ (min)	Band A	Band B	Types of flavonoids^a^
1	19.3	347.0	264.9	Flavones
2	19.9	313.6	256.7	Flavones
3	20.8	352.9	255.5	Flavonols
4	22.7	347.0	264.9	Flavones
5	23.1	348.2	255.5	Flavones
6	26.8	342.2	263.8	Flavones

^a^According to the UV-vis spectra range provided by Tsimogiannis et al. (2007).

The chromatograms also clearly revealed that EADL contains a low number of flavonoid-based bioactive compounds as only six major peaks were detected at the wavelength of 366 nm. Comparison between the chromatograms of several pure flavonoid-based compounds (i.e., fisetin, quercetin rutin, quercitrin, naringenin, genistein, pinostrobin, flavanone, 4′,5,7-trihydroxyflavanone, 2,4,4′-trihydroxychalcone, or dihydroquercetin) against the chromatogram of EADL at 366 nm revealed that peaks at the retention time (R_T_) of 20.1 and 26.8 min represent rutin and quercetin, respectively (Figure 2(c)).

## Discussion

Earlier studies on the hepatoprotective potential of *D. linearis* have revealed the ability of the aqueous, chloroform and methanol extracts of the leaves to attenuate PCM- and CCl_4_-induced liver disease. From the observations using the aqueous and chloroform extracts, it is plausible to suggest that the bioactive compounds responsible for the hepatoprotective activity of *D. linearis* consist of polar and non-polar properties, respectively. However, the ability of methanol extract, which contain polar, non-polar and intermediate polar compounds, to attenuate PCM-induced liver disease seems to suggest that the intermediate polar phytoconstituents might also contributed to the observed hepatoprotective activity. To confirm this, EADL was partitioned from MEDL and subjected to the PCM-induced liver injury tests.

Taking into consideration the involvement of free radical and ROS in the development of PCM-induced liver injury, and the importance of antioxidant restoration to attenuate the PCM-induced hepatotoxicity as described earlier, the first part of the study was carried out to determine the antioxidant and radical scavenging potential of EADL. From the results obtained, EADL was found to contain remarkably higher TPC value, thus, suggesting that it might also possess a high potential to scavenge free radicals and to exert high antioxidant activity. According to Lambert et al. (2007), the phenolic compounds found mainly in the plant-based diet are protective against oxidative damage and adverse effects by ROS and free radicals act as both competent radical scavengers and metal chelators without having to depend on the endogenous antioxidant enzymatic defence system generated by human cells. The finding on high TPC value of EADL was also in accordance with a report by Lai and Lim (2011) on the high TPC content of *D. linearis*. The high TPC value of EADL was similar with the results of phytochemical screening of EADL that revealed the presence of flavonoids and tannins. Sequential analyses carried out on the free radicals scavenging ability of EADL using the DPPH- and SOA-radical scavenging assays confirmed the earlier suggestion that EADL possessed a noteworthy free radical scavenging activity. Based on the reports of the link between TPC value and antioxidant activity (Zheng et al. 2018), the antioxidant capacity of EADL was measured using the ORAC assay and found to be remarkably higher, which corresponds well with the earlier reports. The role of oxidative stress in development of liver diseases has been well acknowledged (Du et al. 2016) and, therefore, it is postulated that any compounds with antioxidative potential are also believed to possess protective effect against liver damage. Based on this postulation, it is reasonable to suggest that the EADL might also possessed the ability to exert the hepatoprotective activity.

Hepatic inflammation is a common trigger of liver disease, and is considered the main driver of hepatic tissue damage. Pu et al. (2016) have demonstrated the critical role of 5‐LOX activity in PCM‐induced liver injury by regulating PCM metabolism and oxidative stress. On the other hand, although allopurinol, an inhibitor of XO, attenuate oxidative stress and liver injury in PCM intoxicated mice, further investigation has disputed the participation of XO in the pathophysiology of PCM-induced hepatotoxicity (Williams et al. 2014). According to Williams et al. (2014), allopurinol-induced liver protection occurs via complicated mechanisms that do not simply involve the ability to inhibit XO or scavenge reactive oxygen, and these might include the adjustment of the intracellular signalling pathways or the up-regulation of the cytoprotective gene expression. In the second part of this study, the ability of EADL to modulate inflammatory process via the LOX or XO pathways was determined using the *in vitro* models. EADL was found to show a very low inhibitory effect against LOX- and XO-mediated inflammatory activity. Hence, it is plausible to suggest that the EADL-triggered attenuation of PCM-induced liver intoxication is not achieved via the inhibition of the LOX- or XO-mediated inflammation. Concurrently, the lack of anti-inflammatory activity via the LOX- and XO-mediated pathway seen with EADL was also reported for MEDL (Zakaria et al. 2017).

In the hepatoprotective study, silymarin, a plant secondary metabolite, was chosen as the positive control drug based on previous report that it has been used worldwide for many years as a complementary alternative medicine because of the beneficial effects associated with the treatment of hepatic diseases (Vargas-Mendoza et al. 2014). This could be associated mainly with silymarin ability to exert antioxidant and anti-inflammatory activities in addition to its anti-fibrotic activity. Interestingly, plant extracts, such as MEDL, have also been reported to exert these activities (Mamat et al. 2013; Zakaria et al. 2017). The hepatoprotective and antioxidant activities of silymarin are caused by its ability to inhibit free radicals that are produced from the metabolism of toxic substances such as PCM, carbon tetrachloride or ethanol. It was also reported that silymarin reversed the PCM-induced liver intoxication by causing significant reduction in the levels of ALT and AST, decrease in the level of MDA while causing significant increase in the activity of SOD and CAT (Kasi Reddy et al. 2017). These biochemical parameter findings were further supported by the histopathological examination. Concurrently, MEDL was also previously demonstrated to exert the similar effects. Furthermore, silymarin was also reported to attenuate PCM-induced liver intoxication via its anti-inflammatory potential, an activity that was also reported for MEDL (Zakaria et al. 2020) and further supported by the absence of inflammation in the PCM-intoxicated liver tissue that was pre-treated with MEDL following the present qualitative histopathological scoring (Zakaria et al. 2017). Since EADL triggers hepatoprotective activity via an similar mechanisms of action that was comparable to silymarin, it is plausible to suggest that *D. linearis* could be potentially developed as an alternative hepatoprotective agent for future human consumption. The fact that silymarin derived from *Silybum marianum* (L.) Gaertn. (Asteraceae), a plant popularly known as milk thistle and found in European and some Asian countries, except Malaysia, plausibly suggests that the former is an expensive source of hepatoprotective agent. Thus, it is beneficial to promote the usage and further develop *D. linearis*, which is wildly grown particularly in Malaysia, as a replacement of hepatoprotective agent that is cheaper than silymarin for use, particularly, in Southeast Asian countries. However, to achieve this, further clinical study needs to be carried out to prove that the product of *D. linearis* is an effective hepatoprotective agent that is safe for human consumption.

Although EADL exerts a weak anti-inflammatory activity via the LOX- and XO-mediated pathways, the significant antioxidant and radical scavenging effects observed seems to suggest that EADL still possesses a capability to exert hepatoprotective activity. Attempts to determine the hepatoprotective potential of EADL against PCM-induced intoxication in rats revealed the ability of EADL to reverse PCM toxic effect on the liver of rats. This claim was based on several observations made during the hepatoprotective study whereby EADL significantly: i) reduced the serum liver enzymes (i.e., ALT and AST) and TB levels; ii) reduced the LW and LW/BW ratio values; which were all found to increase significantly following the intoxication with PCM. The abnormalities induced by PCM intoxication was reversed following the pre-treatment with EADL signifying the partition ability to prevent intracellular enzymes leakage by stabilising or restoring the membrane activity. Moreover, EADL ability to attenuate PCM-induced toxicity in rats is believed to involve the activation of endogenous antioxidant enzymes defence system and attenuation of the lipid peroxidation mechanism based on the observations that EADL: i) increased the activity of liver antioxidant enzymes, namely SOD and CAT, and; ii) reduced the level of MDA, an end product of lipid peroxidation, respectively. The ability of EADL to enhance the activity of SOD and CAT help to neutralise the effect of NAPQ1 and ROS leading to attenuation of liver damage. Interestingly, it was also claimed that natural antioxidants, a characteristic exhibited by EADL as described earlier, strengthen the defence provided by endogenous antioxidant enzymes (i.e., SOD and CAT) from ROS, hence, restore the optimal balance by neutralising reactive species (Azab and Albasha 2018). Interestingly, all of these findings, which lead to a conclusion that EADL possesses the hepatoprotective activity, were further supported by the histopathological findings. From the histopathological analysis, EADL was found to improve the liver cell architectures by reducing the severity of necrosis, promotion of cellular boundaries and protection against broad lymphocytes infiltration. Based on the histopathological findings (Figure 1) and scoring (Table 7), it is worth mentioning that the hepatoprotective effect of EADL occurs in a dose-independent manner. The ability of 50 mg/kg EADL to exert mild necrosis without inflammation, 250 mg/kg EADL to show moderate necrosis and mild inflammation and 500 mg/kg EADL to prevent necrosis and inflammation when compared to the PCM-induced hepatotoxic group suggests the fraction’s dose-independent response. The dose-independent response exerted by EADL could be explained by a phenomenon known as ‘therapeutic window’ wherein certain drugs can only produce suboptimal beneficial effects or even a decline in effects when the dose used was below or above the narrow therapeutic range (Tripathi 2001).

Other than the role of exogenous and endogenous antioxidant pathways, the ability of EADL to exert hepatoprotective activity could also be linked to its phytoconstituents. Qualitative phytochemicals screening of EADL revealed the presence of at least saponins, flavonoids, tannins and polyphenolic compounds. Numerous studies have reported on the antioxidant potential of these classes of bioactive compounds (Chen et al. 2014; Nile et al. 2018; Chai et al. 2019; Kouka et al. 2019). Moreover, saponins, flavonoids and polyphenolic compounds have also been reported to exert hepatoprotective activity against PCM-induced intoxication (Kelava and Cavar 2014; Baali et al. 2016; Huang et al. 2017). Despite no reports on its potential to attenuate PCM-induced liver injury, tannins have also been reported to exert hepatoprotective activity against other inducers (Sobeh et al. 2017). The ability of different classes of bioactive compounds cited above to exert antioxidant and hepatoprotective activities supported the observed antioxidant and heaptoprotective activities of EADL. It is also believed that the hepatoprotective activity of EADL resulted from the synergistic action of different classes of bioactive compounds.

Further analysis of EADL using HPLC revealed the presence of six major peaks at 366 nm, which after the UV-vis spectra analysis revealed the presence of flavonoid-based bioactive compounds. Based on the range of absorbance for Band A and Band B that was characteristics of flavonoids, Peaks 1, 2, 4, 5 and 6 were detected to represent bioactive compounds of the flavones type (Band A falls in the range of 310–350 nm; Band B falls within the range of 250–290 nm) while Peak 3 was detected to represent bioactive compounds of the flavonol type (Band A falls within the range of 350–385 nm; Band B falls within the range of 250–290 nm). Further comparison of the chromatogram that belongs to EADL against the respective chromatogram of several pure flavonoids, namely fisetin, quercetin rutin, quercitrin, naringenin, genistein, pinostrobin, flavanone, 4′,5,7-trihydroxyflavanone, 2,4,4′-trihydroxychalcone, or dihydroquercetin, revealed that only Peak 2 and Peak 6 fall within the exact retention times recorded for rutin and quercetin, respectively. In accordance with this finding, rutin and quercetin have earlier been reported to exert antioxidant (Habtemariam and Belai 2018) as well as hepatoprotective (Janbaz et al. 2002; Barros et al. 2017) activities.

## Conclusions

EADL exerted hepatoprotective activity against PCM intoxication is attributed to its high antioxidant capacity and its ability to scavenge free radicals. In addition, EADL also improves the endogenous antioxidant enzymatic defence system destroyed by PCM overdose, which could be associated with the presence of saponins, tannins, flavonoid-based glycosides and polyphenolic compounds.
